# Dentistry during COVID-19: patients' knowledge and satisfaction toward health protocols COVID-19 during dental treatment

**DOI:** 10.1186/s40001-021-00629-0

**Published:** 2022-01-11

**Authors:** Parvin Parvaie, Freshteh Osmani

**Affiliations:** 1 Dentistry Clinical Research Development Center, Birjand University of Medical Sciences, Birjand, Iran; 2 Infectious Disease Research Center, Birjand University of Medical Sciences, Birjand, Iran

**Keywords:** Patient satisfaction, COVID-19, Knowledge, Dentistry

## Abstract

**Background:**

Coronavirus disease 2019 (COVID-19) as an infectious disease primarily spreading through droplet infection in dental treatment. Patient satisfaction is an indicator of healthcare quality service. Quality of healthcare service and patient satisfaction has been affected by the COVID‑19 pandemic. This study aims to assess the knowledge and satisfaction toward health protocols COVID-19 during dental treatment among dental patients.

**Methods:**

An institutional-based cross-sectional study was conducted on 270 dental patients using a self‑designed questionnaire consisting of knowledge and satisfaction about health protocols COVID-19 during dental treatment through a random sampling technique. Data were imported to SPSS version 21 for analysis. Descriptive and analytical statistics were used to identify the factors associated with their knowledge and satisfaction. A *p* value < 0.05 was considered statistical significance.

**Results:**

Totally, 270 dental patients with mean age of 37.6 ± 6.7 years participated in the study. The mean knowledge score was 36.7 ± 3.5, as considerable number of participants were unaware about the risk associated with dental treatment as well as restrictions imposed on dental procedures. About 18% of participants experienced one or other form of dental complaints during the lockdown period. The overall level of patient satisfaction was 44.6%.

**Conclusion:**

It can be concluded that, public knowledge is to be improved about risk of virus transmission that can be related with dental treatment and also people should be encouraged to use virtual facilities, such as teledentistry, so that no dental emergencies is left untreated during the pandemic time. In addition, the level of satisfaction was in a medium level for dental patients in the study area. Specifically, we deduced from the results that social/physical distancing measures are one of the mechanisms to decrease the fear of exposure to the COVID-19.

## Introduction

Outbreak of COVID-19 began in December 2019 [1, 2]. This virus has spread in the whole world until now. The virus can spread through respiratory droplets, via the mucous membrane of the mouth, nose, and eyes [3, 4]. Using handpieces and ultrasonic instruments during dental procedures leads to the generation of blood and saliva droplets. As a result, these droplets can contaminate the dental instruments and subsequently the surrounding environment. Therefore, both dental practitioners and dental patients could be at risk of being infected [5, 6]. In this way, different studies remarked that dental clinics might be a possible contagion source of viruses. These viruses can be transmitted to the patients and also the practitioner during dental practice. In a dental setting, the risk of acquiring infection from the micro-droplets of an infected patient is high, because the dentist and their equipment are in close vicinity to the patient [1, 7]. The COVID-19 outbreak threatens public health in the world. Its transmissibility is higher than other similar respiratory diseases [8]. To control the spread of COVID-19, there have been adopted some protocols in Iran.

These protocols consist of closing public places, especially dental offices and dental faculties. There is always the worry of experiencing a resurgence in transmission. So that, perhaps, hospitals and clinics are facilitating the transmission of the virus to uninfected patients [9, 10]. The diffusion of aerosol during dental operations puts them at a higher risk of getting infected [11]. Therefore, in some countries, optional dental procedures and oral surgeries have been suspended.

Due to the unique characteristics of dental treatments, standard protective measures in daily clinical practice are not effective enough to prevent the spread of COVID-19. Especially, when patients are in the latent stage of COVID-19 disease and are not aware of their infection or they hide their infection. Therefore, following the principles and health protocols in dental services during the corona epidemic is felt to be more necessary [12, 13]. In addition, measuring the level of awareness of patients referring to the dental clinic to promote the level of awareness of the observance of health instructions in dental service centers is very important. On the other hand, in terms of clinically, evaluating the satisfaction of patients regarding healthcare services is important [14]. In return, the effect of COVID-19 has been reducing procedure and treatment adherence, increase treatment dissatisfaction, and discontinue their treatment follow-up. Since the dental faculties are open based on the decisions made in Iran [15].

Therefore, considering the stated issues and the special importance of observing health protocols to maintain the health of patients and the dental community, this study was aimed to investigate two aspects of awareness and patient satisfaction with the observance of health protocols and instructions related to COVID-19 in the environment of the dental clinic.

## Materials and methods

### Study design

An institutional-based cross-sectional study was conducted from June 10 to 25, 2020, among Iranian dental patients referred to the dental clinic of Birjand city.

The inclusion criteria for this study was having the consent to participate in the study. Questionnaires completed with incomplete information also be excluded from the study.

The referring patients completed the determined questionnaires only if they had consent and preferably one person from each family and for the children, one of the parents answered the questionnaires. In addition, all the explanations about the questionnaires were given to the participants. Finally, after collecting the required questionnaires, all the extracted information was entered into the Spss software, then the data reviewed by the relevant expert for analysis.

### Sample size

The sampling method in this study was easy sampling. According to the sample size formula, a total of 246 samples were obtained, which was considered for this study with a 10% drop in 270 samples. In this research, all patients referred to Birjand dental clinics were examined in the period from the beginning of the study until reaching the required sample size (270).

### Questionnaire

This study was based on a researcher-made questionnaire. In the first stage of implementation of this study, the questionnaire designed and its validation and reliability were evaluated. One questionnaire was prepared to assess patients' awareness of familiarity with health protocols in dental clinics according to the protocols set by the Ministry of Health in dental clinics with the aim of how many the referring people are aware of the observance of these health instructions and to what extent they observe.

Another questionnaire was designed to measure patients' satisfaction with the observance of health protocols in dental clinics according to the Ministry of Health instructions that adjusted by the researcher.

This questionnaire set the principles of satisfaction from the patients' point of view to the principles set by the Ministry of Health. After adjustment, the questionnaires were evaluated for validity and reliability. Questionnaires were provided to 7 experts in the dentistry field to assess its validity. The validity of the questionnaire was calculated using the content validity method. Cronbach's alpha method was also used to measure reliability.

The questionnaire was divided into 3 sections and had a total of 52 questions. The 1st part includes socio-demographic and background information (age, sex, educational level, the continent of residence, and so on), whereas the 2nd, 3rd sections consist of questions to assess the knowledge (23 questions) including three Likert-scale items and satisfaction (17 questions) including five Likert-scale items scored from 1 to 5 (1 = strongly disagree, 2 = disagree, 3 = neutral, 4 = agree, and 5 = strongly agree), of dental patients about COVID-19 health protocols in dentistry, respectively. To calculate the reliability of the questionnaire, a pilot study was done on 30 participants and its Cronbach's alpha (α) was obtained to be 0.78. In addition, the incomplete responses were excluded from the main analysis.

### Statistical analysis

After data collection, they were checked for completeness and coded, cleaned, and then were entered into Spss software version 21. They were analyzed using a *t* test. The Chi-square test or Fisher's exact test also be used to determine the relationships between kinds of variables. All studied variables are first presented using descriptive statistics in terms of data distribution and dispersion. The significance level of the tests was considered less than 0.05.

## Results

A total of 270 dental patients participated in the study and filled the questionnaire with a response rate of 98.6%. Overall, among all the participants, 114 (48%) were male, 126 (52%) were female, and more than half of the participants (*n* = 150, 62%) were between 24 and 35 years and (42.8%) of the respondents were aged more than 45 years. The majority of the study participants were undergraduates or had a secondary school education 174 (64.7% both). The remainder of the participants had completed primary school, middle school, or had a postgraduate degree 29, 26,14 (10.8%, 9.8%, 5.3, respectively). Only 25 (9.4%) participants were illiterate. Among the participants, 122 (45%) were married, and 148 (55%) were single (Table 1).

**Table 1:** Sociodemographic characteristics of study participants

Variables	Number of participants	Percent (%)
Sex		
Male	144	52.4
Female	126	47.6
Age		
15–24	24	8.8
25–34	64	23.7
35–44	67	24.8
> 45	115	42.7
Residence		
Urban	133	49.3
Rural	137	50.7
Level of education		
Primary school	91	22.2
Diploma	212	51.7
Bachelor	46	11.2
Above Bachelor	61	14.9
Marital status		
Married	209	77.3
Single	61	22.7
Family members		
≤ 5	204	75.8
> 5	66	24.2
Occupational status		
Labor	82	30.5
Government employee	67	24.6
Private employee	53	19.5
Housewife	44	16.4
Student	13	4.9
Other	11	4.1
Family monthly income		
≤ 30mRial	79	29.4
30mRial–50mRials	69	25.4
50mRial–70mRials	100	37.0
≥ 70mRials^a^	22	8.2

^a^Million Rials

**Fig. 1:**
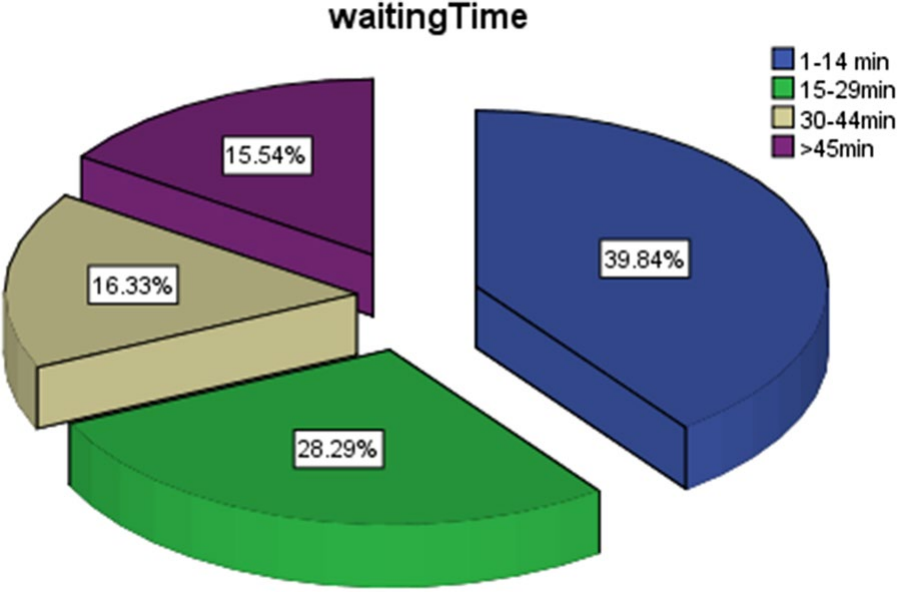
Waiting time to get services and results for participants patients (*n* = 270)

Among all the participants, statements showed that their waiting time as following: less than 15 min: (100 (39.8%)); 15–29 min: (71 (28.3%)); 30–44 min: (41 (16.3%)) and > 45 min: (39 (15.5%)) (Fig. 1). Regarding correct knowledge about the incubation period of the virus, 43.9% (118) answered this correctly (2–14 days), 44.6% (120) selected 7–14 days, 7.4% (20) selected 7–21 days, and 4.1% (12) selected 2–7 days.

### Patients’ knowledge of protective measures and health protocols towards COVID-19 in a dentistry clinic

Knowledge of situations and symptoms that should be considered to identify patients at risk of acquiring the virus, and infection control measures in the dental environment against COVID-19 are summarized in Table 2.

**Table 2:** Correct answers of participants on designed Knowledge questionnaire

Question number	Knowledge on COVID-19	Correct answer (%)
1	It is mandatory to use mouthwash before providing dental services	263 (97.6)
2	During the corona epidemic, all dental services (emergency and non-emergency) should be provided in medical centers	229 (84.8)
3	The use of a sterile disposable package is required to provide all medical services for each patient	226 (83.6)
4	The screening form should be completed by the clients in the clinic	220 (81.4)
	There is nothing wrong with consuming food in the dental environment	
5	It is essential to have a companion in the waiting area of the clinic	221 (82.0)
6	Bringing personal belongings to medical wards is allowed	233 (86.3)
7	Strong and proper ventilation is required in all areas of the clinic medical center (including a reception hall, waiting area, and treatment department)	188 (69.7)
8	The presence of alcohol disinfection gels is necessary to disinfect the hands	252 (93.4)
9	Only the use of a protective mask with a filter mask is sufficient for all medical staff of the clinic	196 (72.5)
10	The use of masks, shields, disposable guns, sleeves, hats, and shoe covers is mandatory for all medical staff of the clinic	223 (82.7)
11	The use of a three-layer mask is mandatory for patients when receiving medical services and attending the clinic	152 (56.2)
12	Mask and shield coverage is mandatory for the dentist and her/his assistant	228 (84.7)
13	The use of an N95 mask is mandatory for patients while receiving medical services	244 (90.3)
14	Only emergency dental treatments should be performed during this pandemic	188 (69.7)
15	Non-emergency dental treatment is essential during this pandemic	154 (57.2)
16	Emergency dental services include toothache, progressive infection control, and control of patients' bleeding, as well as cases that could potentially pose a risk to the patient's health if left untreated	195 (72.3)
17	Covid 19 is transmitted through respiratory droplets	234 (86.7)
18	If the hands are contaminated, the use of an alcoholic disinfectant is sufficient	185 (68.6)
19	Rinsing your mouth before treatment is the most effective way to protect your corona	212 (78.5)
20	Having a shield during the examination is essential for the dentist and assistant	156 (57.7)
21	Having an N95 mask is essential for the dentist and assistant during the examination	246 (91.3)
22	Do you know what the right personal protective equipment is?	169 (62.8)
23	Hand hygiene is no longer required if personal protective equipment is used	156 (57.9)
24	The use of shields (facial protection) is mandatory for the patient when receiving treatment	238 (88.3)
25	The patient must use a filter mask when receiving dental services	254 (94.2)
26	Close contact with an infected person is the most important risk factor for the disease	214 (79.6)
27	Treatments that lead to the production of fine droplets, the use of an N95 mask is mandatory for dentists and assistants	220 (81.7)

*COVID-19* Coronavirus disease 2019

### Knowledge score

The range of knowledge score was between 0 to 46. The mean ± SD of the total knowledge score was 36.7 ± 3.5. Knowledge on familiarity with health instructions of COVID‑19 in the dental environment consisted of 8 questions with a mean ± SD of 9.7 ± 1.7. Knowledge regarding protective measures against corona in the dental environment had 10 questions and its mean ± SD score was 12.87 ± 2.8. In addition, knowledge on the type of dental treatments allowed during the corona epidemic had 5 questions and its mean ± SD score was 6.27 ± 0.9

**Fig. 2:**
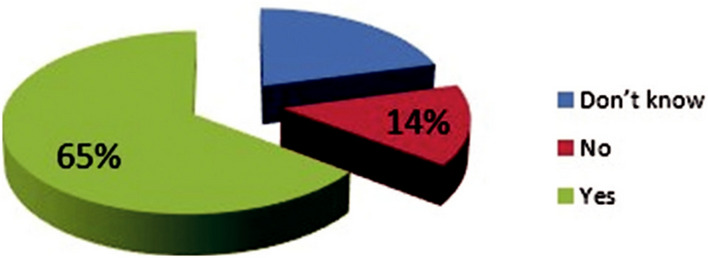
Distribution of patient’s knowledge about protective measures and health protocols towards COVID-19 in dental clinic in Birjand city, (*n* = 270)

The results of this section of the study indicated that 42.4% of dental patients had good knowledge regarding protective measures and health protocols against COVID-19. Although, a significant percentage were unaware of the risk as well as restrictions associated with measures of dental treatment during the pandemic (Fig. 2).

There was no significant difference in their knowledge scores between sex, age groups, and categories of occupation. (*P* < 0.05) (Table 3).

**Table 3:** Association of knowledge on coronavirus disease 2019 with baseline characteristics

Overall score	Mean ± SD	
	36.7 ± 3.5	
Age		
< 30	32.75 ± 7.9	0.93
30–39	40.1 ± 2.8	
40–49	39.1 ± 3.3	
50–59	32.9 ± 1.5	
≥ 60	29.7 ± 6.7	
Gender		
Male	33.4 ± 3.2	0.21
Female	35.8 ± 3.6.6	
Residence		
Rural	37.1 ± 8.1	0.62
Urban	42.7 ± 4.6	
Occupation		
Employed	38.1 ± 2.9	0.77
Self-employed	31.9 ± 3.7	
Unemployed	30.7 ± 6.8	
Student	36.2 ± 5.6	

^*^P < 0.05 significant. *SD* Standard deviation, *COVID-19* Coronavirus disease 2019

### Satisfaction with the implementation of health measures and protocols regarding COVID-19 in the dental environment

Overall, among all of the respondents, 229 (85%) have noticed the availability of hand sanitizer at the dental clinic entrance and disinfect their hands before entry to the clinic. Almost one-third of participants have maintained physical distance at registration and waiting areas. Regarding wearing gloves, masks, and face shields, they have reported 96.5% and 86.5% of dentists wearing gloves with masks and face shields, respectively, while providing healthcare services. Only 39 (14.5%) of the study participants have reported the availability of COVID-19 screening services at the dentistry clinic.

### Patient satisfaction

The overall patient satisfaction was 44.6% with a confidence interval (95% CI 40.1–49.6). Whereas, 55.4% of study participants had dissatisfaction. Regarding patient satisfaction by educational level, more than half of the 48 (53.5%) with primary school education were satisfied. More than two-fifth of the study participants 57 (47.1%) who came from urban were satisfied (Table 4).

**Table 4:** Distribution of satisfaction status by sociodemographic variables

Variables	Satisfied, *n* (%)	Unsatisfied, *n* (%)
Level of Patient satisfaction by educational status		
Primary school	48 (53.5)	42 (46.5)
Diploma	52 (24.6)	160 (75.4)
Bachelor	5 (9.3)	41 (89.7)
Above Bachelor	8 (12.6)	53 (87.4)
Level of Patient satisfaction by monthly income		
≤ 30mRial	54 (68.9)	142 (31.1)
30mRial–50mRials	24 (22.9)	48 (70.1)
50mRial–70mRials	38 (37.7)	62 (62.3)
≥ 70mRials	13 (60.5)	9 (39.5)
Level of Patient satisfaction by Residence area		
Urban	57 (47.1)	64 (52.9)
Rural	79 (53.2)	70 (47.1)

**Fig. 3:**
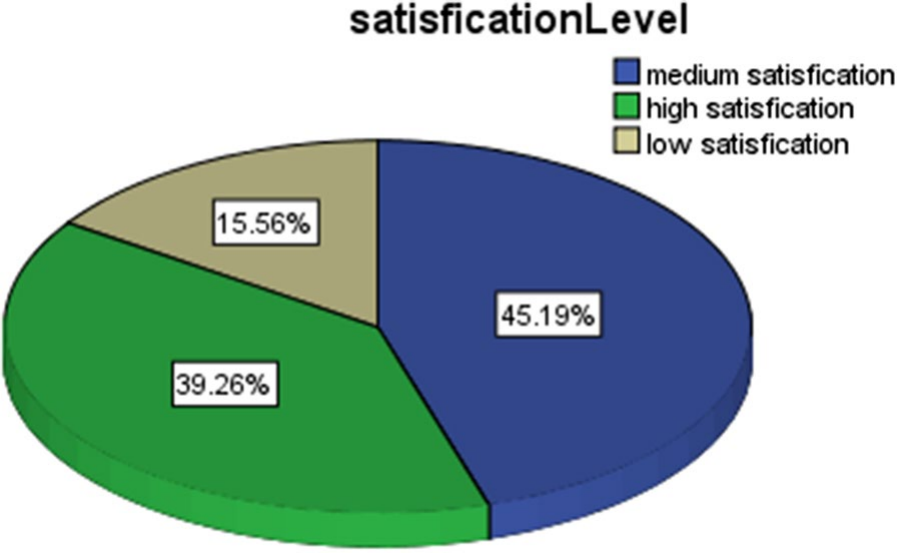
Satisfaction level of patients from the implementation of health measures and protocols regarding COVID-19 in the dental environment (*n* = 270)

Frequency distribution of satisfaction level showed that 106 (39.3), 122 (45.2%), and 42 (15.2%) had high, medium, and low levels of satisfaction, respectively (Fig. 3).

## Discussion

To the best of our knowledge, this is the first survey to evaluate the dental patients’ knowledge and satisfaction regarding the implementation of health measures and protocols regarding COVID-19 in the dental environment along with treatment needs awareness during the pandemic time. All age categories in both men and women were included, with majority groups denoting a good socioeconomic background. Nearly, 65% of the dental patients were found to have sufficient and good knowledge. This relatively high value implies the effectiveness of public information measures and advisories issued by government and health organizations during the COVID‑19 outbreak. Recent researches showed good knowledge in infection control as a predictor of good practice [16, 17]. Using sanitizer and alcohol for hand cleaning at the clinic entrance was significantly associated with patient satisfaction. This may be due to COVID-19 infection prevention. As patients become aware of this strategy and unable to get it in the dental clinics may lead to dissatisfaction.

Patients who maintained and observed better social distancing at the registration and waiting places had significantly more satisfaction. This result is since WHO recognized as physical distancing is one of the COVID-19 control strategies [18]. Hence, patients who found out and maintained social distancing increase their satisfaction. In return, overcrowding induces fear of being infected by COVID-19 that may increase patient dissatisfaction. Although, a substantial number of patients were unaware of ways to prevent the risk of COVID-19 infection transmission associated with dental treatment as well as imposed restrictions on dental procedures during this pandemic. Now, It is believed that respiratory droplets and contact transmission are the main sources of interpersonal transmission of COVID-19; but there are disputes regarding whether this virus can be spread through aerosols. A large number of droplets, splatter, and aerosols could be generated during dental procedures and the routine treatment plan with standard protective measures are not efficient enough to hamper the spread of COVID‑19, particularly increasing in asymptomatic COVID-19 subjects or when they are in the incubation period or intend to conceal their infection [19, 20].

The relatively poor knowledge among dental patients about dental precautions proposes the requirement for awareness in these aspects. Participants in the range 30–50 years had higher knowledge scores, maybe due to higher risk perception of the disease.

In India, the conscious actions taken by the central government have also contributed to decelerating the rate of spread of disease [21]. We should debunk myths and feed fear and misinformation by creating reliable sources of information at each level, within communities.

Most of the participants daresay that dental diseases are not serious as reported by over half of the population. Therefore, their knowledge needs to be enhanced in the same population. In addition, in association with the knowledge score about risk correlated with dental treatment, nearly, 70% stated that only emergency procedures as dental treatment should be done during the time of the pandemic. The governing institutions should monitor trends in local case counts and risk of community transmission, then after observing favorable situation can restart nonemergency procedures. On the other hand, SARS-CoV-2 transmission is highly correlated with Oral and maxillofacial surgery. To decrease the pressure on the healthcare system, it is essential to have an obvious concept for prioritizing procedures in surgeries [22]. In addition, due to transmission patterns can change, dental personnel should stay updated regarding the local transmission trends.

## Conclusions

We attempted to provide a comprehensive inspection of the knowledge and satisfaction of dental patients toward health protocols COVID-19 during the time of the pandemic. The results indicate that most of the participants have sufficient knowledge and are positive in their perspective on the whole on prevailing the pandemic. Although, public knowledge is to be improved about the risk of virus transmission during dental treatment and also people should be encouraged to use virtual facilities, such as teledentistry, so that no dental emergencies are left untreated during the pandemic time.

The level of satisfaction was at a medium level for dental patients in the study area. Social distancing status, availability of alcohol, and sanitizer for hand cleaning at the entrance were factors associated with the satisfaction of patients. Dental clinics need to work on the COVID-19 prevention and control strategy such as maintaining social distance, availing, alcohols, and sanitizers at the clinic entrance, also doing regular satisfaction evaluation, and monitoring feedback regularly.

## Data Availability

The data for this study area cannot be made publically available at present. It will be made available from the corresponding author on a reasonable request.

## Abbreviations

COVID-19: Corona Virus Disease-2019
MPH: Master of Public Health
SPSS: Statistical Package for Social Science
WHO: World Health Organization
